# Identification of novel candidate neural genes for diet-induced obesity in outbred heterogeneous stock rats

**DOI:** 10.21203/rs.3.rs-9695368/v1

**Published:** 2026-05-22

**Authors:** Thu Le, Teresa McGee, Osborne Seshie, Trangdai Bui, Gina Giorgio, Angela Beeson, Benjamin Johnson, Gracie Ang, Oksana Polesskaya, Abraham A. Palmer, William Valdar, Richard Mott, Leah C Solberg Woods

**Affiliations:** 1 University College London, Genetics Institute, London, United Kingdom,; 2 University of North Carolina, Chapel Hill, Department of Genetics, Chapel Hill, North Carolina, USA,; 3 Wake Forest University School of Medicine, Department of Internal Medicine, Section on Molecular Medicine, Winston-Salem, North Carolina, USA,; 4 University of California San Diego, Department of Psychiatry, San Diego, California, USA,; 5 University of California, San Diego, Institute for Genomic Medicine, San Diego, California, USA

**Keywords:** Gene x diet interactions, genome wide association study, obesity, hypothalamus, heterogeneous stock rats

## Abstract

**Background:**

Obesity is caused by genetics, the environment (e.g., diet) and their interactions. The brain, specifically the hypothalamus, plays an important role in obesity, but controlled studies using human brain tissue are not possible. The goal of this study was to conduct a genome wide association study using outbred heterogeneous stock (HS) rats to map genetic loci associated with diet-induced obesity. This was followed by RNAseq in the ventromedial hypothalamus (VMH) to identify candidate causal obesity genes.

**Methods:**

We measured multiple metabolic traits (including fat and lean mass, fasting glucose, insulin, and lipids, glucose tolerance, food intake and activity levels) after long-term consumption of a low-fat (LFD) or high-fat diet (HFD) in 2000 HS rats, split equally by sex and diet. Rats were genotyped using low-coverage whole genome sequencing. RNAseq data was obtained from the VMH of a sub-set of 400 HS rats split equally by sex and diet. We used linear mixed models to detect physiological and expression quantitative trait loci (pQTLs and eQTLs, respectively) in the full dataset and separately by diet and sex. Genes with cis-eQTLs that overlapped pQTLs were assessed as candidate causal genes through mediation analysis. We also identified VMH genes differentially expressed by diet in both sexes, followed by pathway analysis.

**Results:**

We identified 47 pQTLs where six mapped multiple traits, 11 were diet-specific and 13 were sex-specific. We identified nine candidate causal genes, including *Pcare*, *Rbks, Mpv17* and *Gpn1* within a pleiotropic pQTL for multiple adiposity traits on rat chr. 6 and *Tnsfs9* within a pQTL for fat pad weight on rat chr. 9. We also identified *Ccdc77* as a candidate gene within a LFD-specific pQTL for activity levels and *Rtel1* and *Polr3k* within a male-specific pQTL for fatty liver. Genes involved in extracellular matrix and inflammation were dysregulated by diet, particularly in females, while males on HFD showed upregulation of several addiction pathways.

**Conclusions:**

We identified novel candidate genes as neural regulators of diet-induced obesity and related traits and confirmed the importance of accounting for diet and sex in genetic studies of obesity.

## Introduction

Obesity and overweight are major risk factors for multiple diseases including cardiovascular disease, type 2 diabetes, cancer and stroke (1). There has been a steady increase in prevalence of overweight and obesity (2), where current obesity prevalence in the United States reaches 68.6% (based on body mass index (BMI) and anthropomorphic measures such as waist-hip circumference) (3), emphasizing the dire state of health in the US and a critical need for new obesity treatments. Obesity is caused by genetics (4), the environment (5) and their interactions (GxE) (6, 7). Although human genome-wide association studies (GWAS) for obesity-related traits (e.g., BMI and body fat distribution) have identified many genetic effects (8, 9), GxE interactions (10, 11) are more challenging to identify. Most human studies for obesity-related traits that do account for genetic interactions with diet (GxD) either fail to report significant results (12, 13) or lack reproducibility due to insufficient power (e.g., sample size), high heterogeneity, measurement error and lack of proper controls (14, 15).

Obesity is, in large part, a brain disease where neuronal circuits play a role in regulating food intake and peripheral metabolism (16–18). The hypothalamus, a key brain region involved in satiety signaling and energy homeostasis (19–22), is divided into sub-sections (23). The ventromedial hypothalamus (VMH) is known to play an important role in obesity, lipid metabolism, and glucose production and uptake (24–30). The VMH sends (31) and receives (32, 33) signals from the arcuate nucleus, a region well known for regulating satiety signaling via the leptin-melanocortin pathway (34, 35). Although genes located near BMI-associated loci in human GWAS are enriched within the hypothalamus (36, 37), these studies did not account for diet. Importantly, it is impossible to collect human brain tissue under controlled conditions after a specific diet treatment.

Animal models of human complex disease offer several advantages over human studies, including the ability to regulate environmental exposures such as diet and to collect tissues, including brain, under tightly controlled conditions. Our laboratory uses outbred heterogeneous stock (HS) rats for genetic mapping of obesity and other metabolic traits. HS rats were created from eight inbred founder strains (38), with known differences in metabolic traits (39). They have been bred for over 100 generations, approximating the spectrum of common genetic variation segregating in humans (40, 41). Previous work in our lab has demonstrated the utility of HS rats for understanding genetics underlying obesity when rats were on normal chow (42–44) and shown translational relevance to humans (45–47). Here, we conducted a controlled diet study in a large cohort of HS rats to identify genetic loci for obesity/metabolic health, including those that interact with diet or sex. We then assessed gene expression changes within the VMH in response to diet and employed mediation analysis to identify candidate causal genes within the genetic loci. We also identified genes that were differentially expressed by diet in both sexes, followed by pathway analysis.

## Methods

### Animals and Diet

The outbred NIH HS rat colony was initiated in 1984 from eight inbred founder strains: ACI/N, BN/SsN, BUF/N, F344/N, M520/N, MR/N, WN/N, WKY/N, and maintained to minimize inbreeding [13]. Rats used in this study were maintained at Wake Forest University school of Medicine (NMcwiWFsm:HS, Rat Genome Database number 13673907) (41), weaned at 21 days of age and housed 2 rat of the same sex per cage (except for one week for evaluating food intake) in micro-isolation cages (non-siblings housed together) in a conventional facility. Throughout the course of the study, 1942 HS rats were saved from 824 distinct breeder pairs. To maximize genetic heterogeneity, we randomized only one or two males and females from each breeder pair (two males and/or females from 234 breeder pairs, with only one male and female from the other breeder pairs), with siblings placed on different diets. We saved 12 experimental animals each week throughout the course of the study (July 2020 through January 2024). All rats were housed at standard temperature and humidity conditions on a 12-hour light/dark cycle with *ad libitum* access to food and water. Breeders and experimental rats up to five weeks of age were fed standard rodent chow (Lab Diet, Prolab RMH 3000, #5P00). At five weeks of age, experimental rats were randomly assigned to either high fat diet (HFD) or low-fat diet (LFD) (Research Diets #12492: 5.21 kcal/g, 60% fat, 20% carbohydrate, 20% protein; or #D12450J: 3.82 kcal/g, 10% fat, 70% carbohydrate, 20% protein; both diets contain 17% sucrose, and the fat source is lard and soybean oil with all other components being matched). All animal experiments were performed using protocols approved by the Institutional Animal Care and Use Committee at Wake Forest University School of Medicine.

### Study Design

1942 HS rats were phenotyped as shown in Table 1. Each cohort consisted of 12 genetically distinct rats (6 males and 6 females). At five weeks of age, prior to the start of HFD or LFD, baseline adiposity was measured using an EchoMRI. Body weight was then measured weekly. At 12 weeks of age (seven weeks on diet), rats were individually housed for one week to measure food intake. At 15 weeks of age (10 weeks on diet), EchoMRI was used to measure fat and lean mass, followed the next day by an intra-peritoneal glucose tolerance test (IPGTT) to measure fasting glucose, insulin and glucose tolerance. We then implanted a telemeter to measure daily activity. We measured activity when animals were both individually and pair housed. While individually housed, we took a second measurement of individual food intake. Rats were then euthanized after a four-hour fast. Blood was collected for measuring lipids and multiple tissues were collected and snap frozen for subsequent RNA analysis. Spleen was collected for DNA extraction and genotyping. All experiments were conducted at similar times of day across cohorts. Test details are described below. The full list of phenotypes that were collected is listed in **Supplemental Table 1** with raw phenotype data shown in **Supplemental Table 2**.

#### EchoMRI

Prior to diet assignment and after 10 weeks of LFD or HFD consumption, rats went through EchoMRI analysis (EchoMRI LLC, Houston, TX) to measure fat and lean mass. Non-fasted rats were initially weighed and then each rat was scanned in the machine for two minutes in triplicate. EchoMRI occurred in the morning between 9am-12.

#### IPGTT

Rats were fasted overnight for 16 hours. Testing began at 10am +/− 30 minutes at which time fasting blood glucose was measured (Contour Next EZ) and serum was saved for subsequent determination of fasting insulin. We then administered an intraperitoneal injection of glucose (1 mg/kg body weight) and measured blood glucose at 15, 30, 60, 90, and 120 minutes after the injection. Glucose sensitivity was calculated by measuring glucose area-under-the-curve (AUC) where:

$$AUC=\sum \frac{\left(G_{t}+G_{t+1}\right)\times \Delta min}{2}$$

Where *G* is serum glucose levels at time *t*.

Fasting serum insulin was measured with an ultrasensitive ELISA kit (ALPCO, #80-INSRTU-E10).

#### Telemeter implantation for activity measurements

Surgeries took place each week on Thursday. Prior to surgery, rats were given oral meloxidyl (1mg/kg) as an analgesic. Rats were then anesthetized with isoflurane gas. Once fully anesthetized, aseptic technique was used to insert a telemeter (Stellar Telemetry Implant E-430001-IMP-05 X 01, TSE systems) subcutaneously under the rat’s shoulder. Incision was sutured and the rat was placed in a clean empty cage on a warming pad. Once active and ambulatory, the rat was placed into a separate cage. At this time, rats were housed individually for one week to obtain activity and a second measurement of food intake.

Rats were given through the weekend to recover, and telemeters were activated on Monday using Stellar Commander 1.0.0.7/1 software. Activity was recorded while singly housed from Monday afternoon until Friday morning, after which animals were pair-housed with their previous cage mate and activity recording continued until the following Monday afternoon. We thus obtained 3.5 days of activity when individually housed and 3.5 days of activity when pair-housed.

#### Euthanasia and tissue harvest

Rats were fasted around 8am, four hours prior to euthanasia. They were then brought to the euthanasia anteroom 40 minutes prior to euthanasia, which generally started at noon. Rats were euthanized by decapitation and blood was collected in serum separator tubes (Fisher Scientific). We weighed and snap froze multiple tissues including: brain, liver, fat pads (retroperitoneal, omental, gonadal), kidneys, heart and pancreas. Brain was removed and snap-frozen within two minutes of the rat being removed from the cage, while all other tissues were removed and snap-frozen within 10 minutes or less after cage removal.

Prior to freezing, we used visual inspection, employing the following scale to assess degree of fatty liver/damage, as previously described (48):

0 = healthy red coloration, smooth uniform texture
1 = notable yellowing present over 30–75% of the surface area of the liver
2 = liver is uniformly yellowed in coloration
3 = uniform, lighter yellow coloration, visible mild to moderate fibrosis

### Low-coverage whole genome sequencing and imputation

To genotype HS rats, we used low-coverage whole genome sequencing. DNA was obtained from spleens, which were collected postmortem. Detailed protocols for spleen collection, DNA isolation, sample processing, preparing sequencing libraries, and performing the sequencing can be found on protocols.io here: https://www.protocols.io/workspaces/cgord/publications. DNA was extracted using the DNA Advance kit (Beckman Coulter), then sequencing libraries were prepared using the Twist 96-Plex Library Prep kit (TWIST Bioscience). Reads were aligned to the rat reference genome mRatBN7.2 (GCA_015227675.2 GCF_015227675.2) using BWA-mem v0.7.17. Mapped sequences were used to construct haplotypes and impute biallelic single nucleotide polymorphism (SNP) genotypes using STITCH v1.6.6 39 (49, 50). This process resulted in a high-quality set of ~5 million SNPs with imputation quality scores (INFO) > 0.9. The genotypes are deposited in the University of California Library Digital Collection as Heterogeneous Stock (HS) Rat Genotypes, Version 6, https://library.ucsd.edu/dc/object/bb65996027; the genotypes for the animals form this paper can be found by sub-setting for the animals used in this work.

### SNP and Founder haplotype dosage imputation and kinship matrix construction

We then derived a subset of 90,363 high-quality tagging SNPs using PLINK v2 (https://www.cog-genomics.org/plink/2.0/), such that every other SNP was in Linkage Disequilibium (R^2^>0.95) with at least one tagging SNP within a window of 100 SNPs. SNPs with mean allele frequency<0.05 were removed. These sites were used for all subsequent analysis.

### Brain dissections and RNA extraction

The VMH was microdissected from frozen brain of 391 HS rats, split equally by sex and diet following coordinates in the Rat Brain Atlas (51). Rats used for RNAseq were the first rat to be euthanized from the cage (to avoid any transcriptomic changes due to the stress of being alone in a cage for several minutes). These animals spanned the phenotypic spectrum for retroperitoneal fat pad weight (RetroFat). Frozen samples were then sent to Azenta Life Sciences (South Plainfield, NJ) for RNA extraction, library preparation and sequencing as described below.

### Library Preparation with PolyA selection and Illumina Sequencing

Total RNA was extracted using Qiagen RNeasy Plus Universal micro Kit following manufacturer’s instructions (Qiagen, Hilden, Germany). RNA samples were quantified using Qubit 2.0 Fluorometer (ThermoFisher Scientific, Waltham, MA, USA) and RNA integrity was checked using TapeStation (Agilent Technologies, Palo Alto, CA, USA). RNA sequencing libraries were prepared using the NEBNext Ultra II RNA Library Prep for Illumina using manufacturer’s instructions (New England Biolabs, Ipswich, MA, USA). Briefly, mRNAs were initially enriched with Oligod(T) beads. Enriched mRNAs were fragmented for 15 minutes at 94°C. First strand and second strand cDNA were subsequently synthesized. cDNA fragments were end repaired and adenylated at 3’ends, and universal adapters were ligated to cDNA fragments, followed by index addition and library enrichment by PCR with limited cycles. The sequencing libraries were validated on the Agilent TapeStation (Agilent Technologies, Palo Alto, CA, USA) and quantified by using Qubit 2.0 Fluorometer (ThermoFisher Scientific, Waltham, MA, USA) as well as by quantitative PCR (KAPA Biosystems, Wilmington, MA, USA).

The sequencing libraries were multiplexed and clustered onto a flowcell. After clustering, the flowcell was loaded onto the Illumina HiSeq 4000 or equivalent instrument according to manufacturer’s instructions. The samples were sequenced using a 2×150bp Paired End (PE) configuration. Image analysis and base calling were conducted by the Control Software. Raw sequence data (.bcl files) generated from the sequencer was converted into fastq files and de-multiplexed using Illumina's bcl2fastq 2.20 software. One mismatch was allowed for index sequence identification. RNAseq data have been deposited in the Gene Expression Omnibus (https://www.ncbi.nlm.nih.gov/geo/) under accession number GSE330152.

### RNAseq quality control:

We used STAR (v2.6.1a) (52) to align RNAseq reads to the reference Rn6.0, PICARD (v2.5.0) to remove PCR duplicates, and featureCount in the Bioconductor package Rsubread to compute gene-level expression counts, which were later normalized by sequencing depth, gene length, and RNA composition using the DESeq2 BioconductoR package (v1.24.0). We excluded very lowly expressed genes with average reads per sample less than one. The normalized expression levels across individuals for each gene were then transformed using the VST variance-stabilising transformation in DESeq2 (53) before being used for eQTL mapping in Matrix eQTL.

### Differential expression and pathway analysis

Differential expression was conducted using the DESeq2 (53) packages. Unwanted variation was estimated by deriving three “remove unwanted variation or RUV” factors from control genes using RUVSeq (54). These control genes are those not associated with Sex, Diet, or Sex_Diet, as determined by the likelihood ratio test comparing the full model ~ 1 + seqbatch + Sex + Diet + Sex_Diet with the reduced model ~ 1 + seqbatch. Control genes with adjusted p > 0.1 were used to calculate RUVg factors W1, W2, and W3, which explained variation not associated with sequence batch (included as a covariate), sex, diet, or sex by diet interactions.

Differential expression was tested in DESeq2 using the model ~ 1 + W1 + W2 + W3 + seqbatch + Sex + Diet + Sex_Diet. The term Sex_Diet is a factorized term to represent interaction effects by looking at differential expression within groups. Significance of each of the main effects, Sex and Diet, and the interaction effects were assessed using the negative binomial Wald test. Log fold changes (LFCs) for Sex and Diet were shrunken with ashr and reported. Significance was defined as adjusted p < 0.1 after B-H FDR correction. Interaction analyses used the same framework with a factorized Sex_Diet term and four contrasts: M_1 vs M_0, F_1 vs F_0, M_0 vs F_0, and M_1 vs F_1.

Gene set enrichment analysis was performed with clusterProfiler (55) using ranked, nonshrunken, unadjusted LFCs from the full DESeq2 results. Enrichment scores were normalized to generate normalized enrichment scores (NES); pathways with positive NES were labeled activated and those with negative NES suppressed. GSEA using KEGG analysis was run with clusterProfiler::gseKEGG. All recommended parameters were used, except that the minimum number of genes in a set was set to 3 to accommodate lower-powered subset analyses. Dot plots were ordered by adjusted p-value.

In DEseq2, the model is Gene expression ~ Sex + Diet + Sequencing Batch

### Statistical analysis

Statistical analyses largely followed the methodology and code-base in our previous study (43). We briefly summarize the methods here.

#### Kinship matrix construction

We estimated a SNP-based genetic relationship matrix (GRM) *K*_*SNP*_ from the tagging SNPs in PLINK v2.0, which was used for genetic mapping as well as to estimate heritability and genetic correlations for the physiological traits.

#### Phenotype correlations and heritability estimates

Each physiological phenotype was quantile-normalized using the rank-inverse Normal transformation and adjusted for covariates (e.g., sex, diet, batch) if they accounted for more than 2% of the total variance (see **Supplemental Table 1**). The resulting residuals were used for correlation and heritability analysis. Phenotypic correlations between traits were computed as Pearson R statistics of the corresponding residuals. Genetic correlations between traits and the additive heritability of each trait was estimated using GCTA (56) using *K*_*SNP*_.

#### Genetic mapping of physiological traits

GWAS was conducted on all quantile-normalized phenotypes in Supplementary Table 1, including raw tissue weights as well as tissue weights adjusted to body weight (e.g., tissue weight/body weight). Covariates were included explicitly in the genetic mapping (i.e., we did not use residuals). Quantitative trait loci for each physiological trait (named pQTLs in this study) were mapped using the miqtl R package (https://rdrr.io/github/gkeele/miqtl/), employing the GRM *K*_*SNP*_ across the 90,363 tagging SNPs. A genome-wide significance threshold for each trait was computed from 500 permutations; generally the threshold was in the range logP=5–6. QTL intervals were estimated by finding all SNPs with R^2^ >0.6 with the peak SNP of the QTL. The peak SNP within each pQTL was used for subsequent mediation analysis and to estimate founder haplotype effects.

Sex and diet-specific effects on pQTLs were identified by repeating the miqtl mapping for each trait on subsets of rats categorized by sex or diet, using the same covariates as in the miqtl analysis of all the animals. For pQTL that mapped in only a single diet and not in the other diet or in the complete data, we used ANOVA in lme4qtl (57) to check for the Gene x Diet (GxD) interaction in the full data set by comparing the linear mixed models

(a) $$y=X\alpha +g^{\text{peak}}\beta_{G}+d\beta_{D}+gd^{\text{peak}}\beta_{GxD}+e$$

(b) $$y=X\alpha +g^{\text{peak}}\beta_{G}+d\beta_{G}+e$$

where *g*^*peak*^ is the genotype dosage at the peak SNP with effect *β*_*G*_ under the pQTL, *d* is an indicator variable indicating the diet with effect *β*_*D*_ and *gd*^*peak*^*β*_*GxD*_ models the interaction of diet and genotype. The covariance structure of the data is modelled using the GRM *K*_*SNP*_. Those pQTLs for which the p-value of the comparison of the models a vs b is <0.05 are considered as having significant GxD and were designated as diet-specific. A similar process was applied to identify GxS interactions, using sex in place of Diet.

#### Founder haplotype reconstruction and estimation of QTL haplotype effects

Each HS rat chromosome is a mosaic of the original eight founder haplotypes. The haplotype mosaic was inferred probabilistically for all chromosomes in all rats using the hidden Markov (HMM) model in R/qtl2 (58), given the ~90k tagging SNP genotypes previously calculated for the rats and the SNP alleles at these locations known for the founder strains (49). Since the haplotype reconstruction is probabilistic, it provides, for each rat at each location, a set of haplotype pair (diplotype) probabilities. The diplotype probabilities at the peak SNPs of mapped pQTLs and eQTLs were used to estimate the effect of haplotype substitutions (haplotype effects) using Diploffect (59). These estimates and their 95% credible intervals were used to determine if peak SNPs for an eQTL had a strain distribution pattern (SDP) consistent with that of the pQTL.

#### Genetic mapping of expression traits

We mapped eQTLs using MATRIX-eQTL (https://cran.r-project.org/web/packages/MatrixEQTL/index.html) including as covariates sex, diet, technician, sequencing batch and the first principal component of the gene expression matrix, the GRM as the individual covariance matrix. A threshold of logP=5 was employed to call cis-eQTLs within each tissue. Cis-eQTL were constrained to lie within 2 Mb of the corresponding gene location.

#### Mediation analysis and bmediatR

Genes with a *cis*-eQTL overlapping a pQTL were considered as potential mediators of the corresponding physiological trait if the *cis*-eQTL contained at least two associated SNPs within the interval of a pQTL (as defined by *R*^2^ = 0.5). For these potential mediators, we performed mediation analysis, that is, we tested for evidence that the QTL peak (*g*) acts on the physiological trait (*y*) through gene expression (*t*), either wholly (complete mediation) or in part (partial mediation). This was done in two complementary ways: first, using causal steps, as proposed by Baron and Kenny (60) and used in Hong-Le et al. (43), and modeling *g* in terms of SNP genotype dosage; and second, as a verification step, using Bayesian model selection via bmediatR (61), and modeling *g* in terms QTL diplotype probability.

First, for causal steps, consider gene *z* with a *cis*-eQTL mapping within the interval of a pQTL. The evidence for mediation is evaluated by comparisons among three models:

(1) $$y=X\alpha +g^{\text{peak}}\beta +t_{z}\gamma +e$$

(2) $$y=X\alpha +g^{\text{peak}}\beta +e$$

(3) $$y=X\alpha +t_{z}\gamma +e$$

where *y* is the normalized physiological phenotype, *t*_*z*_ is the normalized expression for gene *z*, and *g*^*peak*^ is the SNP genotype dosage for the peak SNP within the pQTL. A Mediator is called when model 1 fits significantly better than model 2 (as assessed by an ANOVA partial F test comparing the models). *P* values of all tested genes within each QTL are corrected for multiple testing using the Benjamini-Hochberg false discovery rate (BH-FDR) (62), with significant mediators defined as genes with q values <0.1. For significant mediators, full mediation is implied when model 1 fails to be significantly more predictive than model 3 (nominal *P* value <0.05); otherwise, mediation is considered partial.

Second, to further understand mediation mechanisms, all significant mediators discovered through causal steps above were tested using bmediatR. bmediatR considers 8 possible models describing relationships between *g*, *t*, and *y*, including models for complete mediation, partial mediation, and “co-local”, where *g* affects *t* and *y* but independently. Giving each model an equal prior probability of 1/8, bmediatR then applies Bayesian model selection to obtain posterior probabilities for each model, as well as Bayes factors, reported here on the log10 scale (log10BF), that summarize the extent to which movement from the priors to the posteriors reflects evidence for mediation of any kind (partial or complete). Since this method was used for verification rather than discovery, the Bayes factor thresholds were lenient: log10BF > 2 indicated strong evidence for mediation; 1 < log10BF ≤ 2 indicated evidence for mediation; 0.5 < log10BF ≤ 1 indicated weak evidence for mediation; values between 0.5 and −0.5 reflect a lack of information either way; and any other negative values were considered evidence against mediation. bmediatR was performed with *g*, the QTL state, defined in terms of either genotype (as allele dosage, one parameter) or diplotype probability (i.e., 36 parameters), with interest focused primarily on the latter, since it provides the most complementary perspective. Moreover, mediation analysis was performed on both the full (unstratified) dataset, with covariates for sex and diet, and on the HFD and LFD groups separately (ie, stratified), with a covariate for sex. All pQTL diplotype, transcript, and phenotype sets with evidence of mediation were reported as strong candidates for further analysis if they had evidence for SNP-based mediation through causal steps and diplotype-based evidence through bmediatR.

## Results

We first investigated the correlation structure and genetic architecture of the obesity related traits listed in **Supplemental Table 1** in 1,942 HS rats, sampled from both sexes and maintained under either HFD or LFD. We then measured gene expression in hypothalamus tissue of 391 of these animals and related it to the physiological data to determine causal pathways mediating genotype to phenotype and diet.

### Adiposity correlates with metabolic health across all groups while correlations with activity levels differ by diet.

**Figure 1.**
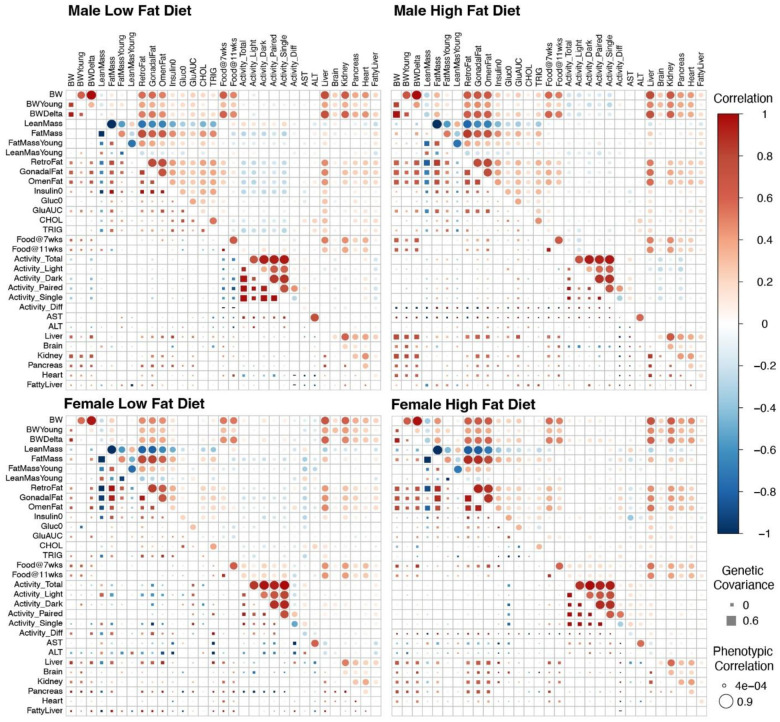
Genetic and phenotypic correlations for physiological traits separated by sex and diet. The upper triangle shows the phenotypic correlations while the lower triangle shows the genetic correlations. Significance levels can be found in Supplemental Figure 1.

As expected, fat mass from the EchoMRI positively correlates and lean mass negatively correlates with individual fat pad weights (r = +/−0.50 – 0.83, p < 0.001) and with other metabolic traits including fasting insulin (Insulin0; r = +/−0.34 – 0.47, p < 0.001), fasting glucose (Gluc0; r = +/−0.13 – 0.30, p < 0.001), serum cholesterol (CHOL) (r = 0.12 – 0.44, p < 0.01) and serum triglycerides (TRIG; r = 0.22 – 0.47, p < 0.001) across all sex and diet groups (Figure 1; **Supplemental Figure 1; Supplemental Table 3–6**). Fat mass also correlates with body weight (BW), with a stronger correlation in rats on HFD (r = 0.32 – 0.43, p < 0.001) than those on LFD (r = 0.14 – 0.26), p < 0.01). Food intake at either 7 weeks, 11 weeks or both correlates with BW (r = 0.49 – 0.61, p < 0.001) and fat pad weights (r = 0.09 – 0.38), independent of sex and diet. Interestingly, food intake positively correlates with lean mass only in rats on LFD (r = 0.14–0.16, p < 0.01), while a negative correlation between food intake and lean mass is seen in females on HFD (r = −0.16), with no correlation between these traits in males on HFD. Correlations with activity levels differ by sex where activity levels negatively correlate with body weight in males on HFD (r = −0.19, p < 0.01) and with fat mass and fat pad weights (r = −0.14 – −0.24, p < 0.05) in males on LFD. There is no correlation between activity levels and body weight, fat mass, lean mass or fat pad weights in females. We also estimated phenotypic genetic correlations, to quantify how much phenotypic correlation is attributable to shared additive genetics, and found they generally mirror the phenotypic correlations, although tend to be stronger in animals on HFD relative to those on LFD.

### Strong heritability of most traits across sex and diet groups

Similar to our previous work, heritability, estimated from the SNP-based additive genetic relationship matrix, is strongest for body weight and fat mass traits, with higher heritability when on HFD (*h*^*2*^ = 0.42 – 0.69) than LFD (*h*^*2*^ = 0.31 – 0.49) (Table 2). Heritability is moderate for metabolic traits (Gluc0, Ins0, CHOL, TRIG, Glucose area under the curve (GlucAUC)), food intake and activity levels, independent of diet (*h*^*2*^ = 0.06 – 0.44). Other than activity during the light period (Activity_Light), activity levels were highly heritable in males on LFD (*h*^*2*^ = 0.5 – 0.61), with moderate heritability in the other groups (*h*^*2*^ = 0.12 – 0.43). Fatty liver was heritable only males (*h*^*2*^ = 0.11 LFD and 0.17 HFD), while serum alanine aminotransferase (AST) was more heritable in females (*h*^*2*^ = 0.30 LFD and 0.27 HFD).

### Differentially expressed hypothalamic genes by diet differ by sex

**Figure 2.**
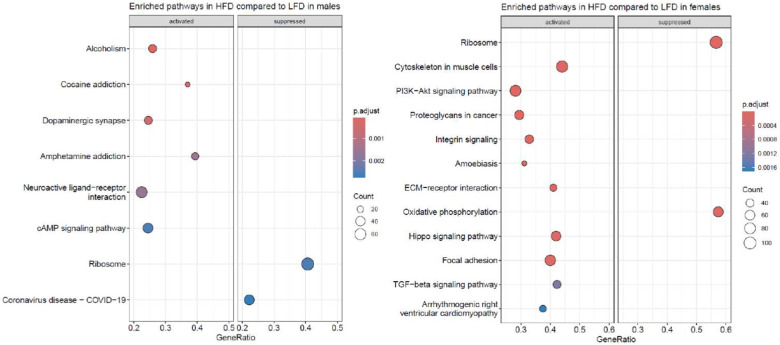
Pathway analysis of RNA sequencing data of the ventromedial hypothalamus showing pathways impacted by high fat diet in male and female HS rats.

We then asked which genes are differentially expressed (DE) in the VMH between diet, as quantified by DESeq2 (53). We identified 101 genes that are DE by diet using the full dataset with sex as a covariate (false discovery rate (FDR) < 0.1; **Supplemental Table 7**). When stratified by sex, many more genes are DE by diet in females (269) than males (11; **Supplemental Table 7**), likely due to higher intrinsic variation in gene expression in the male data. Pathway analysis in the full dataset demonstrates a downregulation of genes involved in ribosome and oxidative phosphorylation, while there is an upregulation of genes involved in the extracellular matrix (ECM; cytoskeleton in muscle cells, integrin signaling, proteoglycans in cancer, focal adhesion and ECM-receptor interactions), PI3K-Akt signaling, inflammation (e.g., TGF-beta-signaling, hippo signaling), and addiction (alcoholism, cocaine addiction, amphetamine addiction) among others (**Supplemental Table 8**). When stratified by sex, oxidative phosphorylation, ECM pathways, PI3K-Ak5 signaling and proliferation/inflammation (Hippo signaling, TGF-beta signaling) are dysregulated in females on HFD, while addiction pathways (alcoholism, cocaine addiction, amphetamine addiction), dopamine synapse and cAMP signaling are altered only in males (Figure 2, **Supplemental Tables 9,10**). Ribosomal genes are downregulated in both males and females.

### Multiple physiological QTL identified in the full dataset

**Figure 3.**
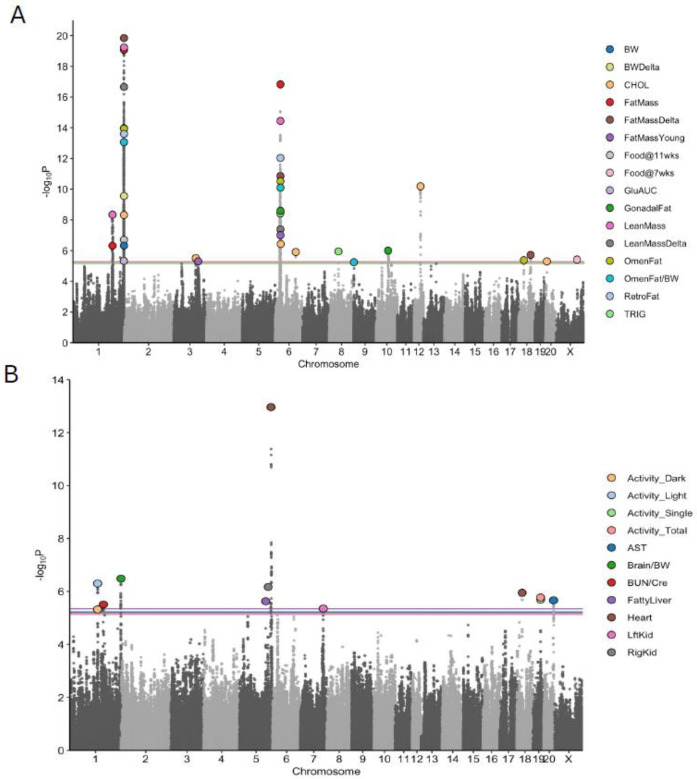
Porcupine plots showing genome-wide scans of multiple metabolic traits using the full dataset. Due to the large number of traits, we show the data in two plots: A) Adiposity, metabolic and food intake traits and B) Activity, tissue weights, and serum biochemistries. The x-axis shows genomic position and y-axis the significance as _log10 of the P value of the test of association. Traits are color-coded, as indicated in the legend. Thresholds for genome-wide significance are shown as horizontal lines and indicate the genome-wide 10% thresholds, as determined by permutation.

We then mapped physiological QTL (hereafter pQTL) associated with the physiological traits, initially using the full dataset (combining sexes and diets) to identify pQTL. We identified 23 pQTL, of which six are pleiotropic, mapping multiple traits (**Table 3;** Figure 3a, b). These include highly significant loci on rat Chrs. 1:259,628,230bp and 6:27,064,422bp that contain multiple adiposity pQTLs as well as LeanMass and LeanMassDelta. The Chr. 1 locus also contains BW, change in body weight (BWDelta), GlucoseAUC, Brain/BW, Food@11weeks, and CHOL while the Chr. 6 locus maps TRIG and CHOL. Activity levels map to two pleiotropic loci: Chr1:141,239,990 for Activty_Light and activity during the dark period (Activity_Dark) and chr19:37,309,444 for total activity (Activity_Total) and activity while house singly (Activity_Single). A pleiotropic locus on chr18:26,161,137 maps omental fat pad weights (OmenFat), BWDelta and Heart. Finally, a locus on chr1:201,383,401 maps both FatMass and LeanMass. 17 pQTL map only a single trait. The average mapping interval (defined as r^2^>0.6) width for all loci is 1.97Mb, ranging from 0.11 – 7.98Mb.

### Identification of diet-specific and sex-specific pQTL

**Figure 4.**
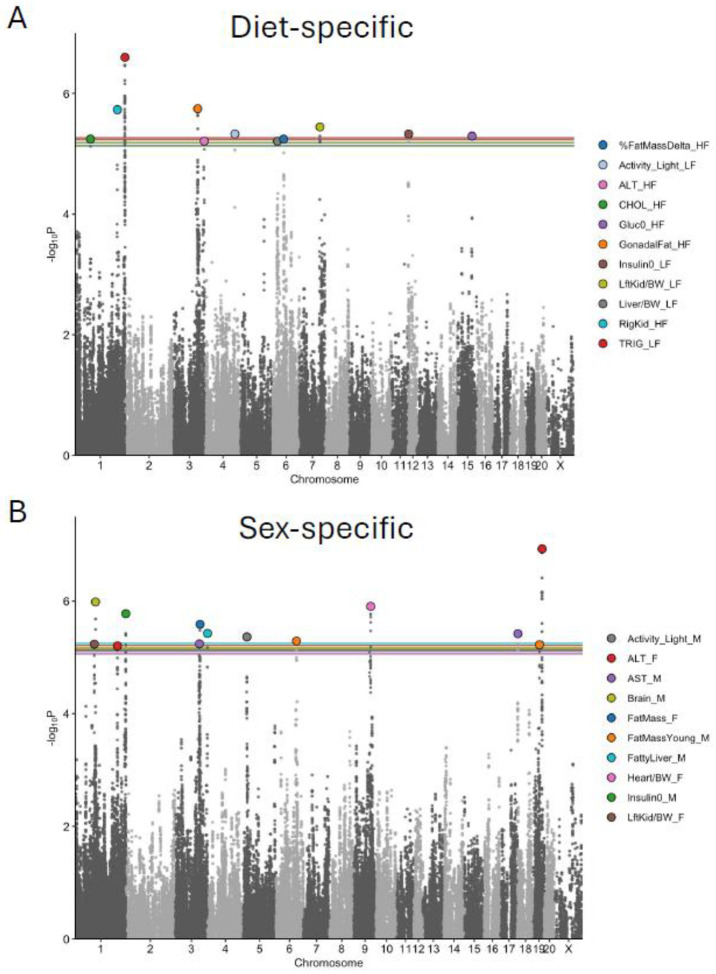
Porcupine plots showing genome-wide scan of multiple metabolic traits that are A) diet-specific and B) sex-specific. The x-axis shows genomic position and y-axis the significance as _log10 of the P value of the test of association. Traits are color-coded, as indicated in the legend. Thresholds for genome-wide significance are shown as horizontal lines and indicate the genome-wide 10% thresholds, as determined by permutation. Only those physiological QTL that are significant after testing for a diet x SNP or sex x SNP interaction using ANOVA are shown (see also Tables 4 and 5).

When the rats are stratified by diet, we identify 11 diet-specific pQTL at 10% FDR: six under HFD conditions (right kidney weight (RigKid) chr. 1, CHOL chr. 1, gonadal fat pad weight (GonadalFat) chr. 3, Serum aspartate aminotransferase (ALT) chr. 3, %FatMassDelta chr. 6, Gluc0 chr. 15) and five under LFD conditions (TRIG chr. 1, Activity_Light chr. 4, liver/bw chr. 6, left kidney weight by body weight (lftkid/bw) chr. 7, Insulin0 chr. 12; **Table 4;** Figure 4a). None of these loci are pleiotropic. The average mapping interval width is 1.76Mb with a range from 0.31 – 3.92Mb.

If instead the rats are stratified by sex, there are 13 sex-specific pQTL: eight in males (Brain chr. 1, TRIG and Insulin0 chr. 1, AST, chrs. 3 and 18, FattyLiver chr. 3, Activity_Light chr. 5, FatMassYoung chrs 6 and 19) and five in females (ALT chrs. 1 and 19, Lftkid/BW chr. 1, FatMass chr. 3, Heart/BW chr. 9; **Table 5**; Figure 4b). One male-specific locus on Chr1:258,530,115Mb maps both Insulin0 and TRIG. Interestingly, this locus encompasses the same confidence interval as the pleiotropic pQTL for multiple BW and adiposity traits on Chr. 1 in the full data. The average mapping interval is 1.46 with a range from 0.23 – 3.28Mb.

### A sub-set of hypothalamic cis-expression QTL are diet-specific

We analyzed 28,213 transcripts with detectable expression levels in the VMH where 4,522 map as cis-eQTL (**Supplemental Table 11**). 66 of these map as diet-specific eQTL (**Supplemental Table 12**).

### Identification of candidate causal genes

To identify candidate causal genes within the pQTL, we first used co-localization to identify cis-eQTL that map within the pQTL. We then used mediation analysis (43, 60) and bmediatR (61), followed by correlation analysis and assessment of the SDP to prioritize candidate genes within each locus. The number of hypothalamus-expressed genes within the pQTL ranged from 3–211 (**Supplemental Table 13–15**). Within the pQTL for the full data, we identified nine full mediators (**Table 6**) which correlate with the trait of interest (**Supplemental Tables 16–18)** and are supported by bmediatR (see **Supplemental Figure 2; Supplemental Table 19**), as discussed below.

**Figure 5.**
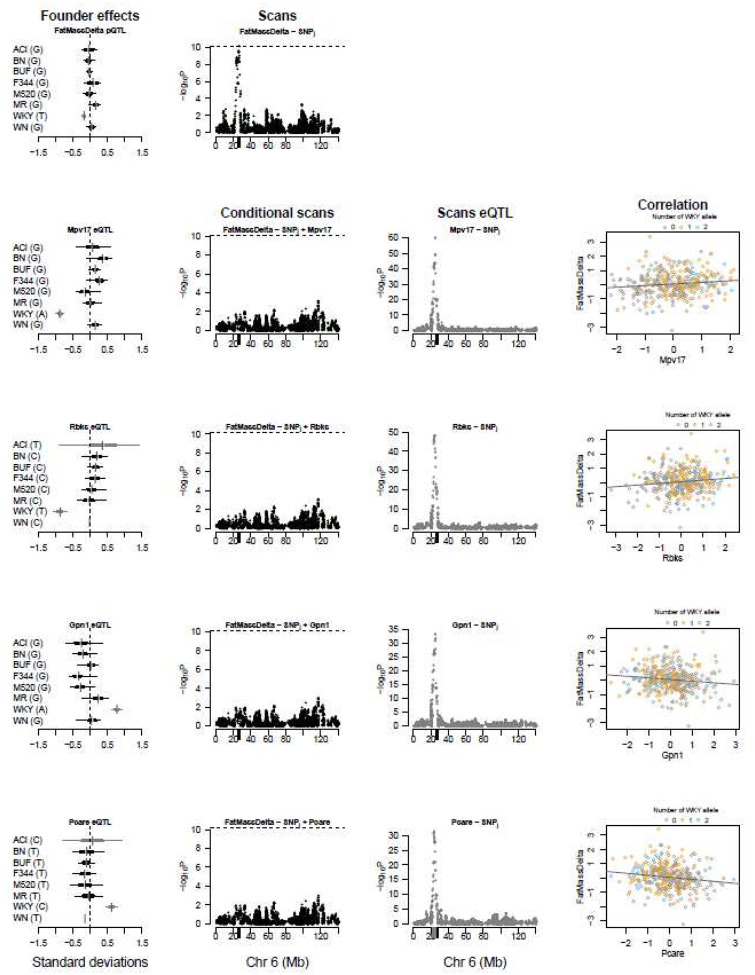
Four genes expressed in the ventromedial hypothalamus (VMH) are identified as mediators within the pleiotropic adiposity locus on rat chromosome 6. The first column shows estimated founder haplotype effects at the peak SNP, shown as box-whisker plots. Note that the WKY haplotype leads to decreased FatMassDelta (change in fat mass from time of diet start), decreased VMH expression of *Mpv17* and *Rbks* and increased expression of *Pcare* and *Gpn1*.

**Figure 6.**
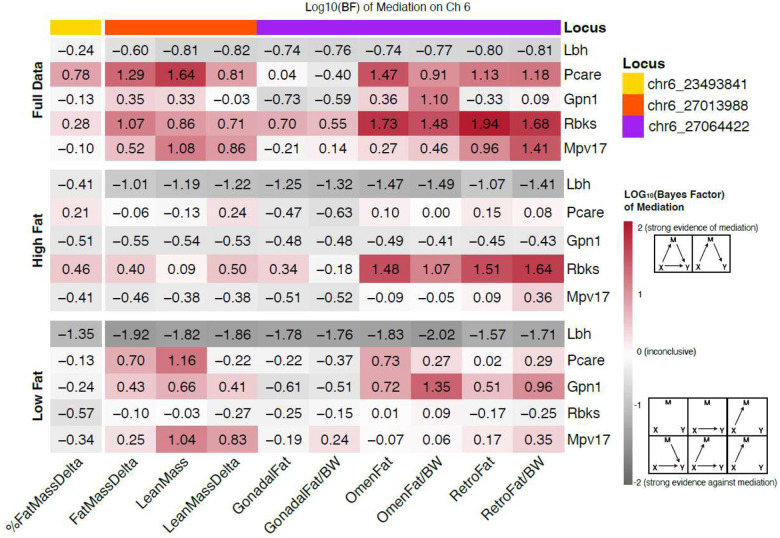
bmediatR results for the pleiotropic adiposity locus on rat chromosome 6. Each table column corresponds to a QTL locus (X) and phenotype (Y) combination, and each row to a gene whose expression level (M) is potentially mediating the effect of X on Y. Each table cell quantifies the evidence for mediation on the log10BF scale, with positive numbers favoring mediation, negative numbers favoring non-mediation, and zero being inconclusive (see scale on right, and Methods). Evidence for mediation is shown for diplotype-based analyses on both stratified (lower two subtables) and unstratified (top subtable) data, illustrating where inferred relationships are the same or different between the HFD and LFD groups. Color coding indicates strength of the mediation where darker red indicates strong evidence of mediation and grey indicates evidence against mediation.

Within the pleiotropic pQTL on rat chr. 6, there were 96 genes, 14 of which map as cis-eQTL within the VMH. Of these, we identified four full mediators (*Photoreceptor cilium actin regulator* (*Pcare), GPN-Loop GTPase 1 (Gpn1), Ribokinase (Rbks)* and *Mitochondrial inner membrane protein MPV17 (Mpv17*)) that correlate with the adiposity traits and are supported by bmediatR (Figure 5; **Supplemental Figure 2**), with the strongest evidence for *Pcare* and *Rbks* (Figure 6). All four genes share the same SDP among the HS founder strains, where the WKY haplotype results in decreased fat mass and either an increase or decrease in gene expression levels (Figures 5). Interestingly, when separated by diet, *Rbks* shows evidence for mediation only under HFD conditions while *Pcare, Gpn1* and *Mpv17* show stronger evidence for mediation under LFD conditions (Figure 6). We then used the transcriptome wide association analysis feature in RatGtex (https://ratgtex.org/), a database that compiles gene expression from HS rats across multiple projects. Expression of all four genes within multiple brain regions strongly associate with adiposity where *Mpv17* and *Rbks* positively associate and *Pcare* and *Gpn1* negatively associate, further supporting our findings here.

No mediators were identified for TRIG or CHOL at this locus indicating that these traits are likely under separate genetic control.

The second column shows the significant peak on rat chromosome 6 (where the dashed horizontal lines represent maximum log(P value) of the physiological QTL), followed by conditional scans where expression levels of each of the four mediators are included in the model. Note that significance level of the FatMassDelta peak drops completely for each of the four genes. The third column shows expression QTL mapping within the VMH for each of the four genes. The fourth column shows the correlation between FatMassDelta vs. gene expression in VMH across 391 rats, using a scatter-plot color-coded by genotype of the WKY allele at the peak SNP of the FatMassDelta pQTL.

**Figure 7.**
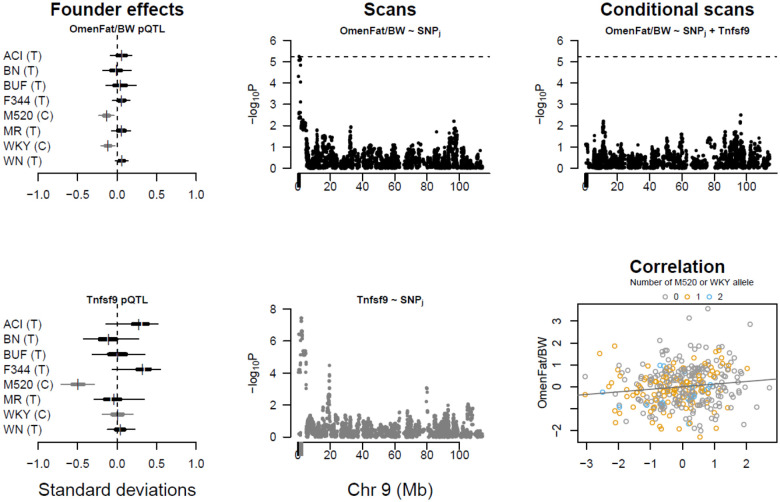
VMH expression of *Tnfsf9*, identified as a full mediator for the Omental Fat Pad Weight over body weight (Omenfat/BW) locus on rat chromosome 9. The first column shows estimated founder haplotype effects at the peak SNP, shown as box-whisker plots. Note that the M520 and WKY haplotypes lead to decreased OmenFat/BW, with the M520 haplotype also leading to decreased VMH expression of *Tnfsf9*. The second and third columns show the significant peak on rat chromosome 9 (where the dashed horizontal lines represent maximum _log(P value) of the physiological QTL), followed by conditional scans where *Tnfsf9* expression levels are included in the model, resulting in disappearance of the peak. Also pictured is expression QTL mapping within the VMH for *Tnfsf9* and the correlation between OmenFat/BW vs. *Tnfsf9* gene expression in the VMH across 391 HS rats, using a scatter-plot color-coded by genotype of the M520/WKY allele at the peak SNP of the OmenFat/BW pQTL.

Within the Chr. 9 locus for OmenFat/BW, there were 44 genes, 11 of which map as cis-eQTL within the VMH. Of these, only one gene, *Tumor necrosis factor superfamily member 9 (Tnfsf9)*, was identified as a full mediator that correlates with OmenFat/BW (r = 0.13, p = 0.01). The M520 and WKY haplotypes share a C at this locus, leading to decreased OmenFat/BW relative to the other founder haplotypes, with the M520 haplotype leading to decreased *Tnfsf9* expression levels and the WKY haplotype not impacting expression levels, at least at the peak marker (Figure 7). *Tnfsf9* is strongly supported by bmediatR (see **Supplemental Figure 2**).

Within the pleiotropic locus on rat Chr. 1 there are 27 genes, five of which map to the VMH. ENSRNOG00000065260 was identified as a partial mediator for BWDelta and Brain/BW, but not the other traits that map to this locus. BmediatR shows strong support for this gene, indicating it is likely a full mediator (**Supplmental Figure 2**).

There were ten genes within the pleiotropic chr. 18 locus for BWDelta, OmenFat and Heart, where only *Epb41l4a* is expressed in the VMH (**Supplemental Table 13**). Although *Epb41l4a* expression levels in the VMH correlate with BWDelta, this gene is not supported as a mediator.

**Figure 8.**
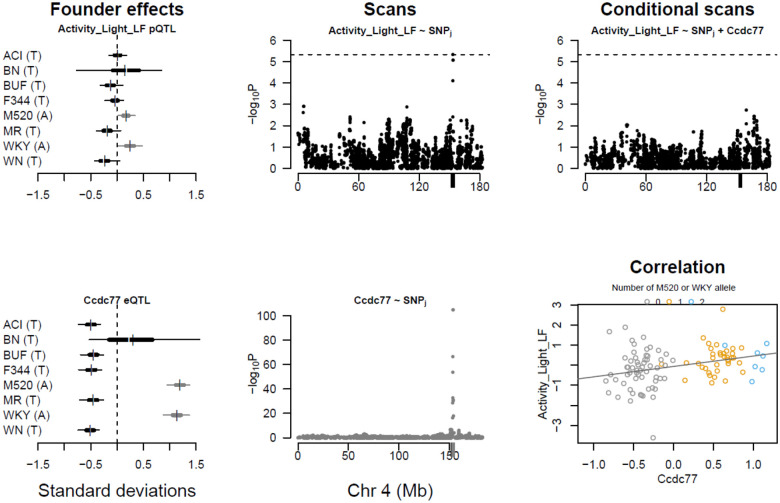
VMH expression of *Ccdc77* is identified as a full mediator for the low-fat diet-specific physiological QTL for activity levels during the light phase (Activity_Light) on rat chromosome 4. The first column shows estimated founder haplotype effects at the peak SNP, shown as box-whisker plots. Note that the M520 and WKY haplotypes lead to increased Activity_Light and increased VMH expression of *Ccdc77*. The second and third columns show the significant peak on rat chromosome 4 (where the dashed horizontal lines represent maximum log(P value) of the physiological QTL), followed by conditional scans where *Ccdc77* expression levels are included in the model, resulting in disappearance of the peak. Also pictured is expression QTL mapping within the VMH for *Ccdc77* and the correlation between Activity_LIght vs. *Ccdc77* gene expression in the VMH using a scatter-plot color-coded by genotype of the M520/WKY allele at the peak SNP of the Activity_Light pQTL.

**Figure 9.**
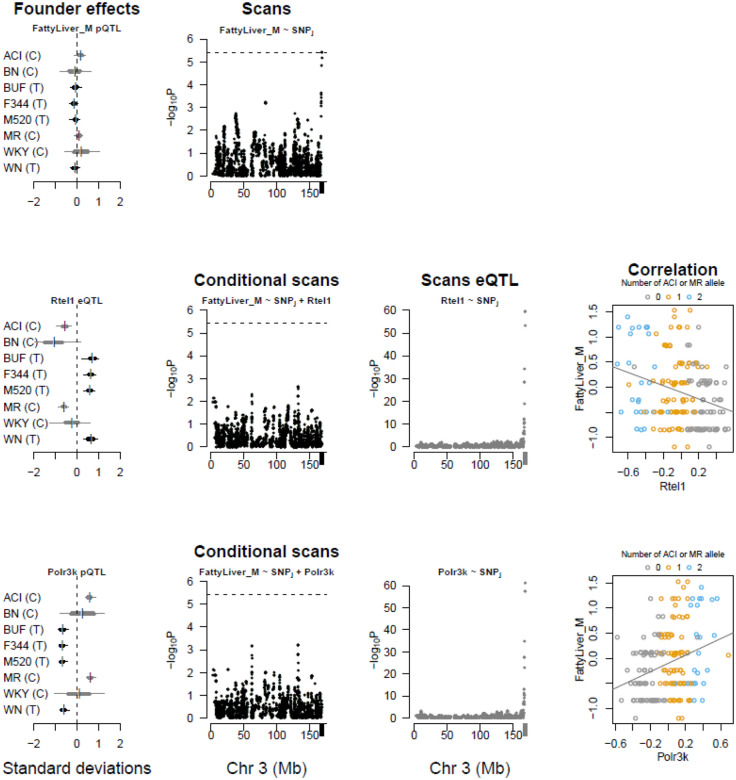
VMH expression of *Rtel1* and *Polr3k* are identified as full mediators for the fatty liver score (FattyLiver) locus on rat chromosome 3. The first column shows estimated founder haplotype effects at the peak SNP, shown as box-whisker plots. Note that the ACI and MR haplotypes lead to increased FattyLiver where BN and WKY share the C allele at this location, but do not seem to impact the phenotype, although large error bars indicate insufficient information. The C allele also leads to decreased VMH expression of *Rtel1* and increased expression of *Polr3k*. The second column show the significant peak on rat chromosome 3 (where the dashed horizontal lines represent maximum log(P value) of the physiological QTL), followed by conditional scans where *Rtel1* and *Polr3k* expression levels are included in the model, making the peak disappear. The third column shows expression QTL mapping within the VMH of *Rtel1* and *Polr3k*. The fourth column shows the correlation between FattyLiver vs. *Rtel1* and *Polr3k* gene expression in the VMH using a scatter-plot color-coded by genotype of the ACI/MR allele at the peak SNP of the FattyLiver pQTL.

Within the LFD-specific locus for Activity_Light on chr. 4, there were 12 genes, six of which map as cis-eQTL (**Supplemental Table 14**). Of these, only *Coiled-Coil Domain-Containing Protein 77* (*Ccdc77*) was identified as a full mediator that also correlates with the trait (0.31, p < 0.01) and is supported by bmediatR (**Supplemental Figure 2**). The M520 and WKY haplotypes at this locus increase activity levels and *Ccdc77* expression levels. (Figure 8; **Table 6**). Within the male-specific locus for FattyLiver on Chr. 3, there were 47 genes; only five map as cis-eQTL within the VMH (**Supplemental Table 15**). Of these, *RNA Polymerase III Subunit K* (*Polr3k*) and *Regulator of telomere elongation helicase 1 (Rtel1*) were identified as mediators that correlate with the trait (r = 0.29, p < 0.01 and r = −0.28, p < 0.01) and are supported by bmediatR (**Supplemental Figure 2**). The ACI and MRI haplotypes at this locus lead to increased fatty liver score, with decreased expression of *Rtel1* and increased expression of *Polr3k* (Figure 9; **Table 6**).

## Discussion

By employing a controlled diet study in an outbred rat model, we identified novel candidate genes within the VMH that may play a causal role in driving obesity. We also demonstrated the role of gene-diet and gene-sex interactions on several metabolic traits and identify candidate genes within some loci. Furthermore, we found that HFD alters the VMH transcriptome, leading to disruptions in oxidative phosphorylation, ECM remodeling, and inflammation. Males, but not females, also show disruptions in addiction pathways.

Our work identifies both novel and known pQTL for multiple metabolic traits. Using mediation analysis and other tools (correlation analysis, bmediatR, SDP, RatGtex), we identify several novel candidate VMH genes that may underlie these pQTL. Specifically, we found four candidate genes (*Pcare, Gpn1, Rbks* and *Mpv17)* as mediators within a pleiotropic locus on rat chromosome 6 for adiposity (including fat mass, lean mass and all fat pad weights) in the full data-set. *Pcare* and *Rbks* show the strongest evidence of association, where *Rbks* plays a larger role under HFD conditions and the other genes may play a larger role under LFD conditions.

Although none of these genes have previously been associated with adiposity (potentially because this is the first genetic study of adiposity to collect VMH under controlled conditions), there are several reasons why these genes are plausible candidates. *Rbks* plays a vital role in the creation of ATP, acting in the first step of ribose metabolism where increased *Rbks* activity leads to decreased cellular ribose. Previous work has shown that increased ribose in the small intestine leads to increased gut motility and resistance to weight gain in mice fed a HFD (63), in support of our work showing a positive association between *Rbks* and adiposity. Other work has shown that, in the liver, *Rbks* associates with Metabolic dysfunction-associated steatotic liver disease (MASLD), a disease strongly linked to obesity (64). *PCARE* is a ciliary protein that is highly expressed in the photoreceptors but also with strong expression in other brain regions including the hypothalamus. Ciliopathies and ciliary genes, particularly those on neuronal primary cilia, are strongly linked to obesity (65), making this a highly plausible candidate gene. *Mpv17* is a mitochondrial inner membrane protein that is involved in mitochondrial DNA replication and function (66). *Mpv17l2*, a related mitochondrial membrane protein, has recently been identified as a potential candidate in human obesity (67). Given this and the key importance of mitochondrial function in obesity (68), this is also a highly plausible candidate. *Gpn1* is involved in gene transcription and DNA repair, playing a vital role in cell function (69). Future work is needed to verify the role of these VMH genes on adiposity. Given that it is impossible to conduct a controlled study using brain tissue in humans, despite a known crucial role for the brain in obesity genetics (36, 37), our findings may prove highly relevant.

Of interest, the chromosome 6 locus has previously been reported (42–44), and our prior work identified and verified two causal genes (*Adcy3* and *Krtcap3*) within this locus. Specifically, we have previously shown that a coding variant within the transmembrane domain of *Adcy3* leads to increased adiposity in a rat model (70), as does decreased whole-body expression of *Krtcap3* (71, 72). Together, this work highlights the complexity of this region, demonstrating that six (or more) genes from various tissues may be playing a causal role in driving increased fat mass within a single QTL.

In addition to replicating the chromosome 6 locus, we also replicate a previously identified locus for RetroFat and TRIG on rat chromosome 1 (42, 44). In the current study, we map multiple additional traits to this locus, including fat mass, lean mass, all fat pad weights, CHOL, brain weight and food intake, showing that this locus drives multiple related phenotypes. Of interest, both TRIG and Insulin0 map to this locus, but only in males, supporting our previous work in males on chow diet (43, 73). This region is 1.99Mb and contains 27 genes. VMH ENSRNOG00000065260 was identified as a mediator for BWGain and Brain/BW, but not the other traits at this locus. Previous work by our group had identified (43) and verified (74) adipose tissue *Grk5* as a causal gene for fat mass and TRIG within this region, indicating that adipose tissue may play a more important role than the VMH at this QTL.

We also identified *Tnfsf9* as a candidate VMH mediator within a locus for omental fat pad weight on rat chromosome 9. *Tnfsf9* is a cytokine that belongs to the tumor necrosis factor (TNF) ligand family. TNFSF9 has been shown to trigger microglia activation, promoting inflammation after acute ischaemic stroke (75). Given that obesity is known to lead to activation of the immune system resulting in a state of chronic inflammation (45), particularly in the hypothalamus where inflammation is known to occur in response to HFD feeding (76), *Tnfsf9* is a highly plausible candidate gene at this locus.

In addition to identifying candidate neural genes for adiposity and other metabolic traits, our work identifies QTL that interact with diet. Interestingly, all adiposity traits (fat mass, lean mass, fat pad weights) have increased heritability when on HFD relative to LFD, with no differences in heritability by diet for the other traits. We identified 11 diet-specific loci, six under HFD and five under LFD conditions. We identified *Ccdc77* as a candidate mediator within a QTL on rat chromosome 4 for Activity_Light (e.g., activity only during the light phase) only under LFD conditions. Although very little is known about *Ccdc77*, a gene located in the centrosome, previous work has found that this gene falls within a locus that has previously been associated with autism spectrum disorder (77), a disorder which can impact general activity. *Ccdc77* gene expression levels are also associated with comorbidities of type 1 diabetes, including hypothyroidism (78), a condition often associated with lethargy. Future studies are necessary to verify the role of *Ccdc77* in activity. The current work is important because although gene-diet interactions are known to influence obesity, human studies for GxD lack reproducibility (14, 15, 79). Furthermore, although previous studies in mice have shown strong gene-diet interactions for obesity traits (80–82), to our knowledge previous mapping studies have considered only one diet condition. Thus, we expect that this study will lay the groundwork for future gene-diet interaction studies in rodents and other models.

In addition to the diet-specific loci, we identified 13 sex-specific QTL (eight in males and five in females), supporting previous work demonstrating robust gene-by-sex interactions in mice (83) and humans (84, 85). Of particular interest, we identified two potential VMH mediators at a male-specific locus on rat chromosome 3 for fatty liver score, a trait that was heritable only in males. Expression of *Rtel1* negatively correlates with fatty liver and expression of *Polr3k* positively correlates with fatty liver. *Rtel1* is involved in the stability, protection and elongation of telomeres and telomere length has previously been associated with non-alcoholic fatty liver disease (86, 87), providing support for this gene. *Polr3k* is involved in gene transcription and has previously been linked to fatty liver disease, but in the opposite direction of our work where a knock-out of MAF1, a global repressor of *Polr3k*, leads to protection against diet-induced obesity and non-alcoholic fatty liver disease (88, 89).

In addition to QTL mapping and gene identification, this work demonstrates that genes involved in oxidative phosphorylation, ECM remodeling, inflammation/proliferation and addiction are disrupted within the VMH under HFD conditions. These pathways are unsurprising and supported by previous work in brain tissue of C57BL/6J mice (90–92). We have also seen disruptions to similar pathways in adipose and liver tissue in HS rats on normal chow with varying degrees of adiposity (45) while others have seen similar changes in adipose, liver and muscle after HFD feeding in baboons (93). Interestingly, when separated by sex, we see that ECM remodeling and inflammation/proliferation pathways are found only in females while addiction pathways, dopaminergic synapse and cAMP signaling were found only in males, implicating alterations to the reward pathway, as previously shown by several studies (94, 95). It is surprising that ECM remodeling and inflammation pathways are unaltered in males and we suspect that this may be due to the larger variation in gene expression levels found in the male population, potentially masking these differences. To our knowledge, this is one of the first studies to look at differences in gene expression in response to HFD separately in males and females, particularly within an outbred population.

Correlation data show that fat mass correlates strongly with metabolic phenotypes across all sex and diet groups, as expected. Food intake positively correlates with lean mass in animals on LFD, but not HFD, indicating that energy consumed is more likely to turn into muscle/bone only when on LFD. We also saw that activity levels positively correlate with lean mass and negatively correlate with fat mass in males on LFD, demonstrating that male rats that move more are leaner with lower fat mass. This relationship was not seen in females.

There are some limitations to this work. Although this study is extremely well-powered to identify general effects, power is lower for diet-specific and sex-specific loci. Thus, future studies will be needed to increase power and verify these findings. Although there is plausible evidence from the literature for most of the candidate genes identified, future studies are required for validation (e.g., knock-down or over-expression strategies). There is a chance that the candidate mediators are not tissue specific and that the findings here represent global gene expression (e.g., it is possible that *Rbks* plays a larger role in the small intestine and *Rtel1* plays a larger role in liver than they do in the brain). That said, previous work looking at RNAseq in liver and adipose tissue of HS rats identified candidate causal genes that differed from those identified here (43, 44), implicating an important role for the current candidate genes specifically in the VMH.

## Conclusions

In conclusion, our work identifies diet-specific and sex-specific genetic loci associated with multiple metabolic traits in an outbred rat model. We used RNAseq from the VMH to identify novel candidate causal genes for multiple traits. These include *Pcare, Rbks, Mpv17 and Gpn1* within a pleiotropic locus for fat mass on rat chromosome 6 and *Tnfsf9* within a locus for Omenfat on rat chromosome 9. We also identified *Ccdc77* as a candidate gene within a LFD-specific locus for activity and *Rtel1* as a candidate gene within a male-specific locus for fatty liver score. We further show that addiction related pathways are found in male, but not females on HFD relative to LFD. Together these results demonstrate the importance of accounting for both diet and sex in genetic studies for obesity. Identification of novel candidate genes within the VMH has the potential to lead to novel therapeutic targets for obesity and emphasizes the importance of controlled studies using brain tissue in the study of obesity.

## Supplementary Material

This is a list of supplementary files associated with this preprint. Click to download.

- Tables36582026.docx
- SupplementalTables5122026.xlsx
- SupplementalFiguresCombined.pdf

Table 3 To 6 are available in the Supplementary Files section.

**Table 3. Phenotypic QTL in the full dataset:** See attached sheet

**Table 4. Diet-specific phenotypic QTL:** See attached sheet

**Table 5. Sex-specific phenotypic QTL:** See attached sheet

**Table 6. List of Mediators in Full and diet or sex-specific loci:** See attached sheet

## Figures and Tables

**Table 1:** Phenotype Timeline

Age (weeks)	Time on diet (weeks)	Test
3	0	Wean
5	0	Weigh, baseline EchoMRI, place on LFD or HFD
12	7	Food intake
15	10	EchoMRI, IPGTT
16	11	Food intake, individual and pair-housed daily activity
17	12	Tissue harvest

LFD: low-fat diet, HFD: high-fat diet, IPGTT: intra-peritoneal glucose tolerance test

**Table 2.** Heritability across sex and diet conditions

	Full data	Males Low Fat Diet	Males High Fat Diet	Females Low Fat Diet	Females High Fat Diet
**BW**	0.46	0.46	0.69	0.31	0.42
**BWYoung**	0.44	0.42	0.45	0.29	0.25
**Heart**	0.24	0.2	0.19	0.15	0.3
**BWDelta**	0.36	0.38	0.6	0.31	0.41
**LeanMass**	0.47	0.48	0.62	0.57	0.59
**FatMass**	0.51	0.54	0.62	0.64	0.59
**FatMassYoung**	0.43	0.36	0.52	0.50	0.2
**LeanMasYoung**	0.34	0.29	0.37	0.4	0.27
**RetroFat**	0.46	0.46	0.65	0.45	0.59
**GonadalFat**	0.47	0.49	0.62	0.46	0.62
**OmenFat**	0.36	0.34	0.5	0.31	0.51
**Insulin0**	0.12	0.21	0.16	0.2	0.06
**Gluc0**	0.25	0.23	0.31	0.23	0.27
**GluAUC**	0.28	0.33	0.38	0.27	0.19
**CHOL**	0.31	0.44	0.27	0.36	0.32
**TRIG**	0.15	0.35	0.2	0.18	0.07
**Fatty_Liver**	0.09	0.11	0.17	0.04	0
**Food@7wks**	0.28	0.2	0.42	0.3	0.24
**Food@11wks**	0.28	0.21	0.25	0.27	0.32
**Activity_Total**	0.26	0.65	0.31	0.29	0.39
**Activity_Light**	0.26	0.26	0.43	0.41	0.38
**Activity_Dark**	0.29	0.61	0.28	0.23	0.23
**Activity_Paired**	0.2	0.5	0.3	0.28	0.36
**Activity_Single**	0.25	0.54	0.21	0.12	0.3
**AST**	0.19	0.06	0.02	0.3	0.27
**ALT**	0.21	0.06	0.2	0.29	0.13
**Liver**	0.41	0.3	0.49	0.29	0.43
**Brain**	0.26	0.26	0.28	0.17	0.1
**Kidney**	0.39	0.35	0.4	0.36	0.36
**Pancreas**	0.12	0.2	0.18	0.07	0.22

## Data Availability

The datasets generated and/or analyzed during the current study are available at the following: Publicly available in a repository: Genotype data are uploaded onto the University of California Library Digital Collection as Heterogeneous Stock (HS) Rat Genotypes, Version 6, https://library.ucsd.edu/dc/object/bb65996027 RNAseq data have been deposited in the Gene Expression Omnibus (https://www.ncbi.nlm.nih.gov/geo/) under accession number GSE330152 and in RatGtex (https://ratgtex.org/). Included in the paper or Supplementary Information: Raw phenotyping data are provided in Supplementary Table 2.

## List of Abbreviations

Activity_Dark: activity during the dark period
Activity_Light: activity during the light period
Activity_Single: activity while house singly
Activity_Total: total activity
ALT: Serum aspartate aminotransferase
AST: Serum alanine aminotransferase
BW: body weight
BWDelta: change in body weight from time of high fat diet start to end of study
BWYoung: body weight at 5 weeks of age
BMI: body mass index
BUN/Cre: Serum blood urea nitrogen/creatine ratio
CHOL: serum cholesterol
DE: differential expression
Gluc0: fasting blood glucose
GlucAUC: Glucose area under the curve during intra-peritoneal glucose tolerance test
GxD: gene by diet interaction
GxE: gene by environment interaction
GonadalFat: fat mass of the gonadal fat pad
GWAS: genome-wide association study
HMM: hidden Markov model
HFD: high fat diet
HS: heterogeneous stock
Insulin0: fasting serum insulin
IPGTT: intra-peritoneal glucose tolerance test
LFD: low-fat diet
LftKid: weight of the left kidney
OmenFat: weight of the omental and mesenteric fat pads
NES: normalized enrichment score
RetroFat: retroperitoneal fat mass
RigKid: weight of the right kidney
RUV: remove unwanted variation
SNP: single nucleotide polymorphism
SDP: strain distribution pattern
TRIG: serum triglycerides
TNF: tumor necrosis factor
